# How Does Awareness Modulate Goal-Directed and Stimulus-Driven Shifts of Attention Triggered by Value Learning?

**DOI:** 10.1371/journal.pone.0160469

**Published:** 2016-08-02

**Authors:** Alexia Bourgeois, Rémi Neveu, Patrik Vuilleumier

**Affiliations:** Neuroscience Department, Laboratory for Behavioral Neurology and Imaging of Cognition, University of Geneva, Geneva, Switzerland; University of Verona, ITALY

## Abstract

In order to behave adaptively, attention can be directed in space either voluntarily (i.e., endogenously) according to strategic goals, or involuntarily (i.e., exogenously) through reflexive capture by salient or novel events. The emotional or motivational value of stimuli can also strongly influence attentional orienting. However, little is known about how reward-related effects compete or interact with endogenous and exogenous attention mechanisms, particularly outside of awareness. Here we developed a visual search paradigm to study subliminal value-based attentional orienting. We systematically manipulated goal-directed or stimulus-driven attentional orienting and examined whether an irrelevant, but previously rewarded stimulus could compete with both types of spatial attention during search. Critically, reward was learned without conscious awareness in a preceding phase where one among several visual symbols was consistently paired with a subliminal monetary reinforcement cue. Our results demonstrated that symbols previously associated with a monetary reward received higher attentional priority in the subsequent visual search task, even though these stimuli and reward were no longer task-relevant, and despite reward being unconsciously acquired. Thus, motivational processes operating independent of conscious awareness may provide powerful influences on mechanisms of attentional selection, which could mitigate both stimulus-driven and goal-directed shifts of attention.

## Introduction

In order to behave adaptively, because of the limited processing capacity of sensory systems, selective attention mechanisms allow the brain to bias perception in favor of salient or significant information in our environment. In this context, attentional mechanisms should allow for the maintenance of goal-directed behavior in spite of distracting events, while at the same time permit the processing of novel, unattended, and potentially important (e.g. dangerous) events [1]. These two distinct attentional functions have been extensively studied using behavioral paradigms and neuroimaging methods [2–5]. Remarkably, recent studies have pointed out that attentional selection might be modulated by emotional or motivational factors (see [6] for a review). Thus, in various paradigms such as Posner cueing task or visual search, involuntary shifts of spatial attention are induced towards the location of irrelevant stimuli when these are associated with threatening [7, 8] or rewarding value [9–13].

Building on previous work investigating the impact of learned reward associations [9–13], we recently demonstrated [14] that high monetary rewards may be a powerful determinant of attentional selection and can mitigate orienting effects induced by both endogenous and exogenous attentional cues. In our study, participants first performed a visual discrimination task where a given color was systematically paired with higher reward outcome on correct recognition trials; and then performed a visual search task where the target location was cued by either a predictive or a non-predictive white flash. Visual search was slowed by the presence of a distractor with the previously rewarded color, regardless of cue validity [14]. In addition, our results suggested that value-based attentional orienting was modulated to some degree by conscious awareness of the reward association, with more slowing on validly cued trials when participants were aware of the previous reward contingency. This finding converge with others [9–11, 13] showing value-based effects on attention without explicit knowledge of the acquired stimulus value.

However, in the latter studies, reward learning occurred while participants were consciously aware of the reward outcome presented with a particular stimulus, and they only ignored their consistent pairing. In this context, there is growing line of research suggesting that even subliminal rewards (presented without being detected) may acquire intrinsic motivational property and subsequently influence cognitive processes ([15], see [16] for a review). Such observations challenge the view that conscious awareness of rewards is necessary to learn from them and boost performance. For instance, Pessiglione et al. [17] demonstrated that monetary rewards could energize behaviour (i.e. increase force production), even when the presentation of the reward cues was subliminal. Moreover, the effect of subliminal motivation was accompanied by an elevated skin conductance response, suggesting greater arousal unconsciously driven by motivational brain systems. On the other hand, Bijleveld et al. [18] developed a solving mathematical task in which participants could earn money when prioritizing either accuracy or speed to solve equations. Supraliminal but not subliminal rewards influenced task strategy, inducing a change in speed-accuracy trade-off. Thus, in this context, awareness of the reward outcome was necessary to influence performance. Another recent study [19] reported an improvement of performances in a mental rotation-task, with both subliminal and supraliminal rewards, but the neural substrate of these two processes was vastly different.

Taken together, therefore, there is still controversial evidence demonstrating the impact of subliminal rewards on behaviour, with possibly different mechanisms underlying these effects when rewards are consciously or not consciously processed. Moreover, much of the extant literature is based on paradigms, in which stimuli that predict reward either supraliminal or subliminal, have an inherent aspect of motivational significance. This fails to differentiate the specific role of reward on attentional selection, specifically outside of awareness from the known role of reward in the strategic establishment of attentional set. Finally, no previous study strictly compared the influence of subliminal and supraliminal reward learning on spatial attentional orienting.

To address these issues, we developed a visual search paradigm to study how subliminal value-based learning can influence attentional orienting and compare such effects on different components of attention. We systematically manipulated goal-directed or stimulus-driven attentional orienting during search, and examined whether an irrelevant stimulus previously associated with a subliminal reward could compete with exogenous and/or endogenous mechanisms of spatial attention. Control experiments allowed us to verify whether these effects were due to the learned association with reward, rather than non-specific familiarity or co-presence with the stimulus carrying reward information.

## Methods

### Participants

Eighteen healthy volunteers (12 women, all right-handed, mean age 25 years, range 22–34) with normal or corrected-to-normal vision, and no history of neurological or psychiatric disorders, participated in the main experiment. Two groups of respectively 17 and 16 new participants (control experiment 1: 9 women, all right-handed, mean age 24 years, range 20–30; control experiment 2: 9 women, all-right-handed, mean age 26 years, range 21–31) also participated in two control experiments (see below). This study was approved by the Neurosciences Cliniques Ethics Committee of the Hôpital Universitaire de Genève (HUG; no 09–316). Written informed consent was obtained for each participant before participation and adhered to the principles detailed in the Declaration of Helsinki.

### Apparatus, stimuli and procedure

#### Association phase

A PC Dell Optiplex 9010 running E-prime software [20] controlled the presentation of stimuli, timing operations, and data collection. Participants sat at approximately 57 cm from the monitor. Each trial began with a white central fixation cross (1° x 1° of visual angle), presented against a black background during an interval randomly ranging from 1000 to 2000 ms. Then, two visual symbols were presented 4.5° to the left and to the right of the fixation cross, during 6000 ms or until a response was made. One symbol was defined as the target and always presented together with one out of seven other symbols (distractors). All stimuli were letters taken from the Agathodaimon font (around 3° x 3° of visual angle), presented upward or downward. Participants were instructed to maintain their gaze at the central fixation cross and to report the orientation of the target (upward or upside-down) with a corresponding key press, as fast and as accurately as possible.

**Fig 1:**
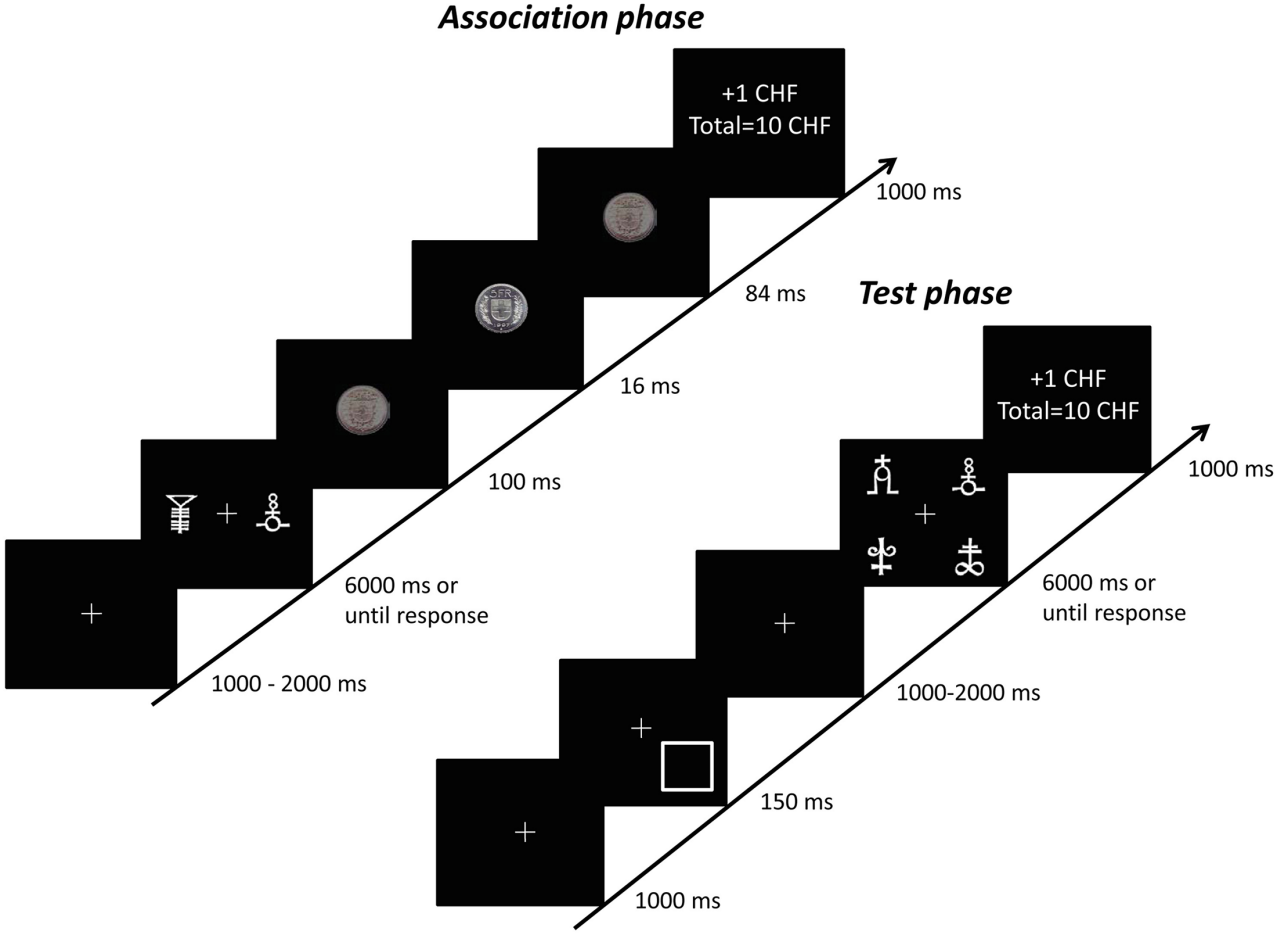
Sequence of events in a given trial. During the association phase, participants were asked to discriminate as fast and as accurately as possible a figure presented either upward or downward. One of the orientation of the figure (counterbalanced across participants) was followed by a high reward (a coin of 5 CHF), or a low reward (a coin of 5 cts) presented subliminally. During the training phase, the target was preceded by a cue, either endogenous or exogenous. In order to investigate the attentional capture of subliminal previously high or low rewarded stimuli, one of the distractors corresponded to the figure associated previously with a high reward on 25% of trials, or a low reward on another 25% of trials. Correct responses were followed by a reward of 1 CHF.

Following previously described procedures (see e.g. [17]), we used subliminal stimulation in order to trigger unconscious processing. Immediately following the response, a reward cue (monetary coin) was briefly flashed during 16 ms in the center of the screen and masked by both forward and backward back disk (Fig 1). A high reward (coin of 5 CHF) was paired with one orientation of the target (e.g. upward), while a low reward (coin of 5 cents) was associated to the other orientation (counterbalanced across participants). This was then followed by a feedback screen (“+1” for correct answer, “+0” for incorrect answer, together with the total number of correct points (correct responses) obtained for the discrimination tasks accumulated over trials. This feedback screen was shown for 1000 ms. Participants were asked to pay attention to the flickering images. This initial acquisition phase consisted of 240 trials (Fig 1).

#### Testing phase

After a short break, the initial association/learning phase was followed by a testing phase. In this condition, a central fixation cross was first presented during 1000 ms. Then, a visual cue consisting of a white empty square (3° x 3° of visual angle) was briefly flashed (150 ms) in one of the 4 quadrant (all equi-probable). The cue correctly indicated the target location on 80% of the trials (valid trials) in the endogenous attention session (predictive condition), and in 50% of the trials in the exogenous attention session (non-predictive condition) [21]. These two sessions were given in counterbalanced order across subjects. After a ranging interval of 1000–2000 ms, the target display was then presented (for 6000 ms or until response), in which 4 different stimuli were placed at equal distance around the fixation cross (centered at 3° of visual angle). Participants had to discriminate a new Agathodaimon letter target, presented upward or downward, always accompanied by three distractors. To probe for the attentional capture by previously high or low rewarded stimuli, one of these three distractors corresponded to the target previously associated with a low or a high reward during the initial association phase (50% of trials each). In the remaining 50% of the trials, distractors were never paired with a reward and therefore referred to as neutral distractors.

Responses were followed by a feedback screen (“+1” for correct answer, “+0” for incorrect answer, together with the total number of points accumulated across trials). Each session included 240 trials (Fig 1 and S1 Table).

#### Control experiment 1: no reward

To control for effects of familiarity as opposed to reward history on subsequent attention orienting, a similar experiment was given to a second group of participant, using exactly the same design but now removing the masked coins of 5 CHF or 5 cents occurring during the association phase. All other aspects were identical.

#### Control experiment 2: stimulus specificity and validity

The above control experiment rules out the possibility that our data may be accounted for by a familiarity effect. However, this manipulation does not allow us to conclude that the interference induced by a high or a low-associated reward is truly related to reward history rather than to previous pairing / co-occurrence with another stimulus, unrelated to reward. Indeed, high and low reward conditions did not differ in our main experiment, but both differed from the neutral condition where the distractor has not been paired with any subliminal stimulus in the learning phase. Moreover, in the main experiment, high and low rewards were associated with the same symbol, but presented either upward or downward. It could thus also be possible that reward history was averaged across these two orientations of the target. We therefore ran another control experiment, using the same design described in the main experiment but with the following exceptions. In the first association phase, one symbol was paired with a subliminal high reward (a coin of 5 CHF), while another symbol was paired with a subliminal neutral, non-rewarded stimulus (a grey circle). This manipulation allowed us to test whether our results might be accounted for by reward history or by the mere association with another stimulus, unrelated to reward. We also used two different symbols paired with either a high reward or a neutral stimulus, rather than only one symbol presented upward or downward in order to prevent reward history from being averaged across the two paired stimuli.

A second limitation of our main experiment was addressed in this further control experiment. In the design used above, exogenous attentional mechanisms were probed with visual cues whose validity probability was 50%, vs 50% for invalid cues. Given that our experiment was composed of 4 locations, it might be possible that some top-down strategic mechanisms might still operate in this exogenous orienting, thus inducing relative changes rather than absolute /categorical differences in attentional mechanisms. Therefore, in this second control experiment, exogenous cues were associated with a probability of 25% for valid conditions and 25% for each of the other 3 invalid locations, in order to maximize the automatic attentional capture induced by attentional exogenous cues.

#### Awareness Check task

At the end of the two phases of the main experiment, we explicitly asked participants if they have seen anything other than the mask during the association phase. In order to verify unawareness of the masked rewards during the association phase, we also administered a control task of 15 trials after the previous two phases. Six trials with masked coins (either 5 CHF or 5 cts) presented during 16 ms, 6 trials with supraliminal coins (either 5 CHF or 5 cts) presented during 500 ms, and 3 catch trials composed of a black screen (instead of the coins) presented for 500 ms between the two masks, were shown. Participants were required to give a forced-choice response in order to indicate if they have seen a coin of 5 CHF, a coin of 5 cts, or nothing during the presentation of each stimulus. Then, they also rated their answer on a confidence scale from 0 (not at all confident) to 5 (very confident). This task was given to all participants tested in the main reward experiment above.

## Results

Only correct responses were included in the analysis. Responses below 100 ms or above 1500 ms were also eliminated as outliers. These exclusions accounted for 3.45% of the trials in the association phase and 2.52% of trials in the training phase. Two participants were excluded because of a very high rate of incorrect responses (>2.5 SD from the participants’ mean data in at least one of the conditions).

### Association phase

Mean RTs to report the target were submitted to a repeated-measures analysis of variance (ANOVA) with the within-participant factor of subliminal reward (high, low). There were no significant differences in RTs to report a high-reward target (651 ms) compared to a low-reward target (658 ms), *F*<1.

### Testing phase

The critical test concerned any slowing of attention orienting toward the search target in the presence of a previously rewarded distractor. A repeated-measures analysis of variance (ANOVA) on mean RTs was performed with the factors of attentional orienting condition (endogenous, exogenous), cue validity (valid, invalid), and reward condition (neutral distractors, previously low-rewarded distractors, previously high-rewarded distractors). The analysis revealed a main effect of validity, F(1,16) = 44.98, MSE = 44526, *p* = 0.001, and a significant interaction between attention and validity, F(1,16) = 21.05, MSE = 12190, *p* = 0.001. Participants were faster to respond to valid compared to invalid trials, especially when attention was endogenously oriented (*ps* < .001).

**Fig 2:**
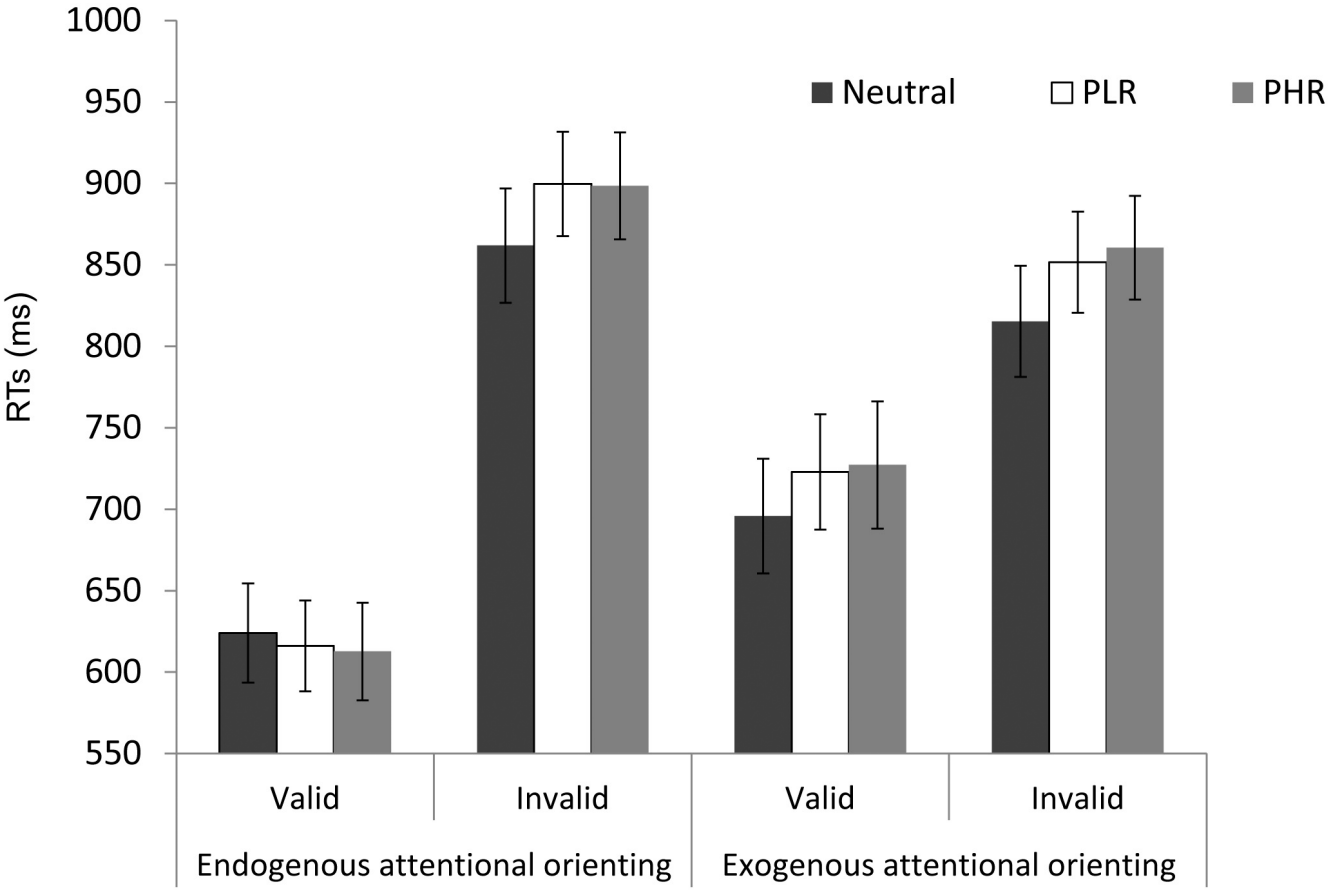
Mean correct RTs (in ms) for endogenous and exogenous attentional orienting as a function of validity (valid, invalid trials), and distractors (neutral, previously low-rewarded; *PLR*, previously high-rewarded; *PHR*). Error bars represent standard errors.

Importantly, the analysis also demonstrated a main effect of reward, F(2,32) = 7.50, MSE = 1810, *p* = 0.002. RTs to targets were slower when a previously high- or low- rewarded distractor was presented compared to when a neutral distractor was presented (*p* = .004, and *p* = .009, respectively). The interaction between validity and reward was also significant, F(2,32) = 3.58, MSE = 1366, *p* = 0.040. Fisher’s LSD post-hoc analysis indicated that participants were slower to respond to invalid targets when a previously high- or low-rewarded distractor rather than a neutral distractor was presented (all *ps* = .001). Thus, previously high- and low-rewarded stimuli involuntarily captured attention, but more strongly so when attention had to be reoriented (Fig 2). There was no significant effect of reward history on valid trials (all *p*s>.27). There was no significant difference between high and low rewards in this analysis (all *p*s>.67).

### Control experiment 1: no reward

In the main experiment, targets were always presented in the association phase together with one distractor, either high or low rewarded. An alternative account for our finding during the testing phase might therefore be that the previously high- and low-rewarded distractors could interfere with performance because of a familiarity effect (due to previous exposure) rather than because of value-based learning. To rule out this account, we tested another group of participants with the same design but without masked coins of 5 CHF or 5 cents during the association phase. Responses below 100 ms or above 1500 ms were also eliminated as outliers (2.16%). We again performed an ANOVA on mean RTs obtained during the testing phase with the factors of attention task (endogenous, exogenous), cue validity (valid, invalid), and distractor type (corresponding to upward target in the association phase, downward target, or neutral distractors). The analysis revealed a main effect of validity, F(1,16) = 101.68, MSE = 15662, *p* < .001, with faster RTs for valid trials (732 ms) compared to invalid trials (878 ms), again mainly when attention was endogenously oriented (interaction attention x validity, F(1,16) = 45.12, MSE = 3411, *p* < .001). More importantly, neither the main effect of reward nor the interaction between reward and validity was significant (*F*<1, mean RTs for upward target, downward target, neutral distractors in valid trials, 729 ms, 743 ms, 724 ms; in invalid trials, 878 ms, 894 ms, 862 ms, respectively).

### Control experiment 2: stimulus specificity and validity

In order to further confirm that the interference induced by a high or a low-associated reward is truly related to reward history rather than to previous pairing / co-occurrence with another stimulus, unrelated to reward, another control experiment was performed. Neutral distractors were now also paired with a non-rewarded subliminal cue during the association phase. We also used two different symbols paired with either a high reward or a neutral stimulus, in order to prevent reward history from being average across the two paired stimuli. Eventually, we used exogenous cues with a probability of 25% for valid cues, instead of 50% of probability in the main experiment, in order to maximize the automatic attentional capture induced by attentional exogenous cues. Responses below 100 ms or above 1500 ms were eliminated as outliers. Two subjects were discarded from this analysis because of abnormally slow RTs (>3 SD from overall mean).

We first performed an ANOVA on mean RTs obtained during the test phase with the factors of attention task (endogenous or exogenous), cue validity (valid or invalid), and distractor type (previously high-rewarded or previously associated with a neutral stimulus). This analysis indicated a significant main effect of validity, F(1,13) = 34.32, MSE = 16848, *p* < .001 with faster RTs for valid (744 ms) compared to invalid trials (888 ms), especially when attention was endogenously shifted (interaction task x validity, F(1,13) = 59.87, MSE = 3723, *p* < .001). There was however no significant modulation of these factors by distractor type, *F*<1. Notably, the proportion of incorrect responses was much higher in this control experiment than in our main experiment (12% of outliers for the exogenous condition, 5% for the endogenous condition), which might reflect individual differences between groups. We therefore performed another ANOVA on accuracy with the same intra-participant factor. This analysis indicated a main effect of validity, F(1,13) = 7.99, MSE = 40, *p* = .014, with more accurate responses on valid (95%) compared to invalid trials (91%). The interaction between validity and distractor type was marginally significant, F(1,13) = 3.98, MSE = 24, *p* = .067. After invalid cues, participants were more accurate in the presence of a distractor previously associated with a neutral (93%) compared to a distractor previously associated with a high reward (90%, *p* = .007). No difference between neutral and previously high rewarded distractors was observed for the valid conditions (95%, 94%, respectively, *p* = .50).

In order to better take into account these differences in error rates in the analyses, we used an index of behavioral efficiency that combines RTs and accuracy (mean RTs/proportion correct for each condition and each subject), allowing reliable comparisons across conditions with different performance levels [22]. This efficiency index was then submitted to a similar ANOVA as above, with the factors of attention task (endogenous or exogenous), cue validity (valid or invalid), and distractor type (previously high-rewarded or previously associated with a neutral stimulus). This analysis indicated a main effect of validity, F(1,13) = 28.34, MSE = 5.32, *p* < .001, with faster RTs for valid (890 ms) compared to invalid (1122 ms, *p* < .001) cues, especially when attention was endogenously oriented, interaction task x validity, F(1,13) = 6.23, MSE = 2.01, *p* = .027. Interestingly, the interaction between validity and distractor type was now also significant, F(1,13) = 7.95, MSE = 0.75, *p* = .014. Participants showed slower RTs for invalid conditions, when a previously high-rewarded distractor (1163 ms) rather than a neutral distractor was presented (1080 ms, *p* = .0.042). No significant effect of distractor type was seen for valid conditions (886 ms, 893 ms, respectively, *p* = .78). The other main effects or interactions were not significant, *F*>1. These results confirm that reward history does interfere with performance, and more strongly so when attention has to be reoriented than when validly oriented by the preceding cues. Remarkably, this interference occurred even when attentional capture was concurrently modulated by purely exogenous spatial cues.

Finally, to further confirm that previously rewarded stimuli do mitigate stimulus-driven shifts of attention for exogenous cues associated with different probabilities (probability of 50% in the main experiment, probability with a 25/25/25/25 split in the Control Experiment 2), we performed another ANOVA on mean RTs while keeping the same intra-participant factor but adding the factor Experiment as a between-participant factor. This analysis did not indicate however significant interactions involving the Experiment factor (all *p*s>.26).

### Awareness control task

All participants reported not seeing anything other than the mask in the first association phase. In order to confirm the lack of awareness of masked rewards during the association phase, we calculated the proportion of seen and unseen trials when coins (either 5 CHF or 5 cts) were presented supraliminally, i.e. during 500 ms, or subliminally, during 16 ms, in the post-testing awareness check phase. Two participants were discarded from this analysis because of the presence of unreliable responses in supraliminal conditions (many “unseen” responses).

On average, participants reported 89% (SD = 25%) of coins presented supraliminally. This was significantly higher than chance (*t*-test = 7.87, *p* < .001). Their confidence rating in this condition was high (average 96%—SD = 5.64%). In contrast, the mean percentage of detected coins, when they were masked, was 59% (SD = 37%), with a confidence rate of 79% (SD = 21%). This was not different from chance (*t*-test = 1.48, *p* = .159). Finally, participants correctly answered in 83% (SD = 20%) of the trials when no coin was presented, with a confidence rate of 82% (SD = 26%).

We next verified whether any individual variability in coin detection would lead to differential reward learning effects. Detection accuracy in the subliminal coin condition from each participant was correlated with the magnitude of their RT slowing in the presence of previously rewarded stimuli in the attention testing phase (RT on trials with high and low reward distractors minus neutral distractors), for both attention conditions separately. No significant correlation was found, neither for the exogenous (rs < .14; *p*s>.59) nor for the endogenous (rs < .37; *p*s>.16) conditions. Hence participants who showed the strongest impact of acquired reward associations at test were not those who tended to more often detect the masked coins during subliminal presentation.

Overall, these results indicate that our paradigm effectively induced subliminal processing of rewards.

## Discussion

Selective attention, either goal-directed or stimulus-driven, provides a mechanism that bias neuronal activity in order to represent behaviorally salient or relevant stimuli, amongst other distracting information competing for awareness. This mechanism is fundamental for survival and adaptive behavior. A similar modulation of attention can arise from emotional or motivational signals, including rewards [6, 9–12, 23]. However, still little is known about how these reward modulations compete or interact with subcomponents of spatial attention, particularly in situations when these effects occur involuntarily and/or outside of awareness. Here we designed a novel visual search task, in which goal-directed or stimulus-driven attentional orienting could be systematically manipulated, and examined whether a stimulus previously associated with a subliminal (low or high) reward compete with these two different types of orienting. Our results demonstrated that irrelevant, but previously high or low rewarded stimuli involuntarily captured attention, leading to slower detection of the search target, especially when attention had to be reoriented (i.e., when such distractor occurred with an invalid spatial cue). Hence, even though the subliminal reward information was never consciously perceived, it was extracted by the brain nonetheless and used to guide attentional orienting in the subsequent test phase.

These results accord with, but also extend studies demonstrating the impact of subliminal reward on performance, as previously observed in simple motor force production and more complex cognitive tasks [17, 18, 24–26]. More specifically, our results reveal a powerful role of reward on both endogenous and exogenous shifts of attention, even when orienting in space is concurrently modulated by another highly salient visual cue. This suggests that these different sources of attentional selection might operate through a common priority map integrating several different top-down influences [27], and so even when reward learning occurring outside of awareness. This dovetails well with results observed using a similar attention design but with supraliminal probabilistic rewards [14]. In the latter study, stimuli previously associated with a high monetary reward were capable of involuntarily capturing attention, despite endogenous or exogenous orienting to another location. However, participants who were aware of the reward contingency showed greatest distraction by rewarded stimuli on valid trials specifically, suggesting that reward awareness may generate more explicit attention strategies and interfere with orienting on such valid trials. In the present experiment, an equivalent attentional capture was also induced by both previously high and low reward but following subliminally acquired information about the association with a particular visual symbol. This suggest that reward processing and learning may still arise even without amplification and integration of the value information with the target representation, as otherwise proposed for consciously perceived stimuli [28].

The neural substrates of unconscious reward learning and visual association remain poorly known, although they likely involve interactions between reward circuits in basal ganglia and attentional systems in fronto-parietal areas [29–34], or at the sensory level in early visual areas [35, 36]. Reward signals have also been observed in lower-level orienting circuits in superior colliculus, which is densely connected to the basal ganglia where reward information is thought to be encoded (see e.g. [37, 38–41]). It is thus possible that value representations, either supraliminal or subliminal, might be encoded in the basal ganglia, in good keeping with other subliminal learning effects involving subcortical structures [17], and then projected to cortical areas in frontal or parietal lobe to dynamically modify behaviour.

These results provide new insights on the functional links between shifts of attention, value-based attentional orienting, and consciousness. Stimulus-driven and goal-directed shifts of attention may be counteracted by reward information, even in the absence of reward awareness. Reward could thus have a direct impact on perceptual representation, independently of its well-known role in the strategic establishment of attentional set [42–44], even when presented outside of awareness. However, it remains possible that although similar behavioral results are obtained both with subliminal and supraliminal rewards, the underlying neural circuit might be different for consciously and non-consciously acquired reward associations. Thus, Bijleveld et al. [19] demonstrated that supraliminal and subliminal rewards improved performances to the same extent. Nonetheless, they found that only supraliminal but not subliminal high value reward engaged brain areas typically involved in reward processing, such as the ventral striatum. Future studies should therefore further dissect the neural bases of reward-based effects on attention and better delineate the different processes recruited by conscious or non-conscious learning.

More generally, such results also open interesting perspectives for rehabilitation strategies of attention disorders, particularly in neglect patients. This syndrome typically occurs after fronto-parietal damage and corresponds to a loss of awareness for stimuli located on the controlesional left hemispace. A recent study [45] demonstrated that the spatial orienting in these patients could be modulated by the presentation of rewarded stimuli on the neglected left hemispace, even if patients had no conscious awareness of any spatial asymmetry in reward delivery. Interestingly, Della Libera & Chelazzi [46] demonstrated in healthy participants not only that attentional processes are influenced by rewards but also that this effect is long-lasting. Another recent study [47] demonstrated with naturalistic stimuli that the effect of reward on perceptual representations can generalize from the level of shape features to the level of conceptual object category. These findings open interesting perspectives for the use of motivational cues such as reward for the rehabilitation of patients with attention disorders.

By establishing the potential role of reward in capturing attention, even when learning occurs outside of awareness, or data suggest that a better comprehension of these mechanisms might be useful to elaborate more efficient rehabilitation techniques in brain-damaged patients suffering from attention disorders.

## Supporting Information

S1 Table Mean correct RTs (in ms) for endogenous and exogenous attentional orienting as a function of validity (valid, invalid trials), and distractors (previously high-rewarded; *PHR*, previously low-rewarded; *PLR*, neutral). Standard Errors are reported in parenthesis.
(TIF)
