# Supplementary figures and images for: How Does Awareness Modulate Goal-Directed and Stimulus-Driven Shifts of Attention Triggered by Value Learning?
Source: PLoS One. 2016 Aug 2;11(8):e0160469. doi: 10.1371/journal.pone.0160469 (PMC4970812; doi:10.1371/journal.pone.0160469)

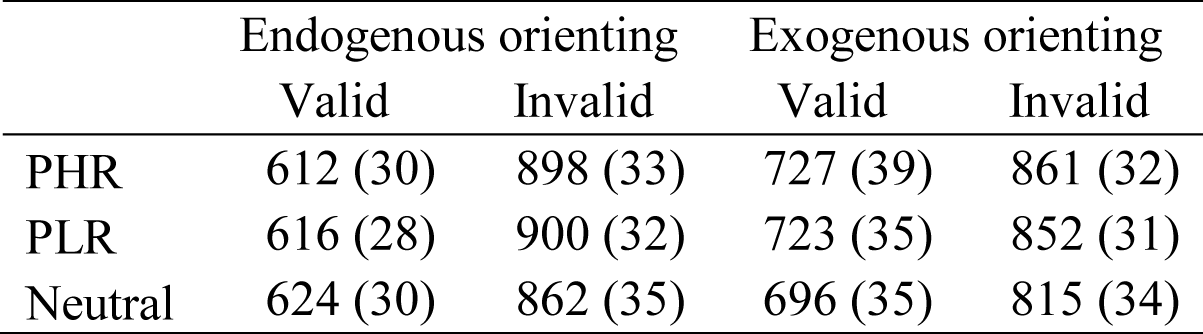

Supplement: S1 Table — Standard Errors are reported in parenthesis. (TIF) [file pone.0160469.s001.tif]
